# Overview evidence on interventions for population suicide with an eye to identifying best-supported strategies for LMICs

**DOI:** 10.1017/gmh.2015.27

**Published:** 2016-02-12

**Authors:** A. Fleischmann, E. Arensman, A. Berman, V. Carli, D. De Leo, G. Hadlaczky, S. Howlader, L. Vijayakumar, D. Wasserman, S. Saxena

**Affiliations:** 1 Department of Mental Health and Substance Abuse, World Health Organization, Geneva, Switzerland; 2 Department of Epidemiology and Public Health, National Suicide Research Foundation, University College Cork, Cork, Ireland; 3 American Association of Suicidology, USA; 4 National Centre for Suicide Research and Prevention of Mental Ill-Health, Karolinska Institutet, Stockholm, Sweden; 5 Australian Institute for Suicide Research and Prevention, Brisbane, Australia; 6 University of Melbourne, Melbourne, Australia; 7 SNEHA, Voluntary Health Services, Chennai, India

**Keywords:** Evidence-based, intervention, interventions, low- and middle-income countries, suicide, suicide attempt

## Abstract

Globally, over 800 000 people died by suicide in 2012 and there are indications that for each adult who died of suicide there were likely to be many more attempting suicide. There are many millions of people every year who are affected by suicide and suicide attempts, taking into consideration the family members, friends, work colleagues and communities, who are bereaved by suicide. In the WHO Mental Health Action Plan 2013–2020, Member States committed themselves to work towards the global target of reducing the suicide rate in countries by 10% by 2020. Hence, the first-ever WHO report on suicide prevention, Preventing suicide: a global imperative, published in September 2014, is a timely call to take action using effective evidence-based interventions. Their relevance for low- and middle-income countries is discussed in this paper, highlighting restricting access to means, responsible media reporting, introducing mental health and alcohol policies, early identification and treatment, training of health workers, and follow-up care and community support following a suicide attempt.

## Background

Globally, over 800 000 people died by suicide in 2012, according to World Health Organization (WHO) Global Health Estimates (WHO, 2014*a*, *b*, *c*). This corresponds to a global age-standardized suicide rate of 11.4 per 100 000 population; 15.0 and 8.0 per 100 000 for males and females, respectively. There are indications that for each adult who died of suicide there were likely to be many more attempting suicide (De Leo *et al*. 2005; WHO, 2014*a*). Taking into consideration the family members, friends, work colleagues and communities, who are bereaved by suicide (Pitman *et al*. 2014), there are many millions of people every year who are affected by suicide and suicide attempts (Berman, 2011).

Notably, suicide is the second leading cause of death in 15–29-year olds (WHO, 2014*a*); for young girls, 15–19 years old, it is the first leading cause of death globally (WHO, 2014*d*). Against preconceptions, 75% of all suicides occur in low- and middle-income countries (LMICs). In more affluent countries, three times as many men die of suicide as do women, but in LMICs the male-to-female ratio is much lower at 1.5:1. Globally, suicides account for 50% of all violent deaths in men and 71% in women. Despite a drop in the estimated global age-standardized suicide rate between 2000 and 2012, that may partially be explained by an improvement in global health, regionally there have been increases in LMICs in the African Region and among men in LMICs in the Eastern Meditteranean Region, emphasizing the need to concentrate and prioritize suicide prevention efforts in LMICs (WHO, 2014*a*). With regard to age, suicide rates are highest in persons aged 70 years or over for both men and women in almost all regions of the world. However, in some countries, suicide rates are highest among the young (Rezaeian, 2008). Young adults and elderly women in LMICs have much higher suicide rates than their counterparts in high-income countries, while middle-aged men in high-income countries have much higher suicide rates than middle-aged men in LMICs. Pesticide ingestion, hanging and firearms are among the most common methods of suicide globally; many other methods are used based primarily on their ready accessibility with the choice of method often varying locally.

In 2013, WHO Member States committed themselves, in the WHO Mental Health Action Plan 2013–2020 (WHO, 2013), to work towards the global target of reducing the suicide rate in countries by 10% by 2020. Hence, the first-ever WHO report on suicide prevention, *Preventing suicide: a global imperative*, published in September 2014 (WHO, 2014*a*), is a timely call to take action using effective evidence-based interventions.

## Evidence-based interventions and their relevance for LMICs

Positive outcomes for prevention most likely derive from strategies involving comprehensive, multisectoral participation, involving health care, education, employment, social welfare, justice, agriculture, nongovernmental organizations, community organizations and others working together in a coordinated manner. One of the most challenging findings of the WHO report was the large variation in the rates and demographic characteristics of suicide across regions and between countries. Therefore, it is likely that a range of different types of interventions will be needed to tailor strategies to each country's cultural and social context. Comprehensive strategies in high-income countries have demonstrated the additive and synergistic effects of integrating multiple interventions (WHO, 2014*a*); this approach could be replicated in LMICs taking into consideration varying cultural contexts. The quality and availability of data on suicide and suicide attempts in many countries, particularly LMICs, is often limited, thus, identifying evidence-based interventions that reduce suicides in LMICs is particularly difficult. Several strategies effectively employed in high-income countries may also be effective in LMICs, but the evidence supporting this assumption is often absent. Very few suicide prevention interventions have undergone rigorous evaluation in LMICs.

For the interventions presented in the following, the evidence is consolidated at the WHO mhGAP Evidence Resource Centre (WHO, 2015).

### Restricting access to means

Direct access or proximity to means (including pesticides, firearms, heights, railway tracks, poisons, licit and illicit drugs, sources of carbon monoxide such as car exhausts or charcoal, and other hypoxic and poisonous gases) is a major risk factor for suicide. The availability of and preference for specific means of suicide also depend on geographical and cultural contexts (Mann *et al*. 2006; Ajdacic-Gross *et al*. 2008).

Restricting access to the means of suicide is effective in preventing suicide – particularly impulsive suicide – as it gives those contemplating suicide more time to reconsider and allows time for intervention and change of mind. While mental disorders are diagnosed in around 90% of suicide cases in high-income countries, psychological autopsy studies in China and India revealed only 40–60% with a psychiatric diagnosis, placing an added importance to means restrictions (Vijayakumar & Rajkumar, 1999; Phillips *et al*. 2002). Implementation of strategies to restrict means can occur both at national level, through laws and regulations, and at local level, for instance by securing risk environments.

Pesticides account for an estimated one-third of the world's suicides (Gunnell *et al*. 2007*a*). Reducing access to pesticides could significantly impact on reducing impulsive suicide in relevant LMICs. Suicide by intentional pesticide ingestion primarily occurs in rural and agricultural areas of LMICs in Africa, Central America, South-East Asia and the Western Pacific. Measures proposed to prevent suicide by pesticides include: ratifying, implementing and enforcing relevant international conventions on hazardous chemicals and wastes; legislating to remove locally problematic pesticides from agricultural practice; enforcing regulations on the sale of pesticides; reducing access to pesticides through safer storage and disposal by individuals or communities; and reducing the toxicity of pesticides (Gunnell *et al*. 2007*b*; Vijayakumar *et al*. 2013). In addition, the medical management of those who attempt suicide by self-ingestion of pesticides should be optimized, particularly through reduced barriers to immediate care (WHO, 2008; Knesper, 2011; NICE, 2011).

Suicide by firearms is a highly lethal method, accounting for the majority of suicides in some countries, such as the USA (Brent & Bridge, 2003; Miller *et al*. 2013). Available data show a close correlation between the proportions of households owning firearms and the proportion of firearm suicides (Anglemyer *et al*. 2014). Legislation restricting firearm ownership has been associated with a reduction in firearm suicide rates in many countries, including Australia, Canada, New Zealand, Norway and the UK.

Historically, intentional carbon monoxide poisoning has been one of the most common methods of suicide in some countries. Legislative and pragmatic changes to domestic gas at national and regional levels have substantially reduced suicide by this method. Collectively, evidence indicates that reducing the lethality of carbon monoxide has a direct effect on decreasing overall suicide rates. Charcoal-burning poisoning is a recent method of suicide by toxic gas that has rapidly become a common method in some Asian countries. Removing charcoal packs from open shelves into a controlled area in major store outlets in China, Hong Kong SAR has significantly reduced charcoal-related suicide deaths (Yip *et al*. 2010).

In most European countries, self-poisoning with medication is the second or third most common method of suicide and suicide attempts (Hegerl & Wittenburg, 2009). Restricting access to and availability of medications that are commonly used in suicide has been shown to be an effective preventive measure (Hawton *et al*. 2013). Health-care providers can play a critical role by restricting the amount of medication dispensed, informing patients and their families about the risks of overdose or prescribed medications, and stressing the importance of adhering to prescribed dosages and disposal of unused tablets.

One central problem in implementing means restriction strategies is that very few LMICs have accurate information on the methods used in suicides and suicide attempts. These methods often vary by geographic region (urban *v.* rural), gender, and age group, and, as the example of charcoal burning in China, Hong Kong SAR indicates, preferred methods can change rapidly over time. Targeted means restriction interventions require simultaneous monitoring of the methods employed in suicidal behaviour to ensure that the most common methods are being addressed, to monitor possible substitution when access to one method is restricted, and to adapt strategies as preferred methods of suicide change.

### Responsible media reporting

Inappropriate media reporting practices can sensationalize and glamourize suicide and increase the risk of ‘copycat’ suicides (imitation of suicides) among vulnerable people. Responsible reporting of suicide in the media has been shown to decrease suicide rates. Important aspects of responsible reporting include: avoiding detailed descriptions of suicidal acts, avoiding sensationalism and glamorization, using responsible language, minimizing the prominence and duration of suicide reports, avoiding oversimplifications, educating the public about suicide and available treatments, and providing information on where to seek help. Media collaboration and participation in the development, dissemination and training of responsible reporting practices are also essential for successfully improving the reporting of suicide and reducing suicide imitation (Pirkis, 2009). These improvements were demonstrated in Australia and Austria following active media involvement in the dissemination of media guidelines (Bohanna & Wang, 2012). Common sense suggests that responsible media reporting about suicide would be effective in all jurisdictions, but further evidence is needed internationally to confirm the value of this type of intervention for reducing suicides in LMICs.

### Introducing mental health and alcohol policies

Whilst mental health should constitute a priority for all governments, suicide prevention efforts should be broadened beyond improving the recognition and treatment of mental disorders. For example, the role of alcohol and substance use disorders in the etiology of suicidal behaviour has traditionally received much less attention than that of other types of mental disorders such as depression, but it has become increasingly evident that alcohol and drugs are important preventable risk factors for suicide in countries and demographic subgroups within countries where alcohol and drug use are common.

The WHO Mental Health Action Plan 2013–2020 (WHO, 2013) and the WHO Global strategy to reduce the harmful use of alcohol (WHO, 2010*a*) provide frameworks and guidance on getting started. The former has the four key objectives of: (i) strengthening effective leadership and governance for mental health; (ii) providing comprehensive, integrated and responsive mental health and social care services in community-based settings; (iii) implementing strategies for promotion and prevention in mental health; and (iv) strengthening information systems, evidence and research for mental health. The latter offers ten policy options and interventions: (a) leadership, awareness and commitment; (b) health services’ response; (c) community action; (d) drink-driving policies and countermeasures; (e) availability of alcohol; (f) marketing of alcoholic beverages; (g) pricing policies; (h) reducing the negative consequences of drinking and alcohol intoxication; (i) reducing the public health impact of illicit alcohol and informally produced alcohol; and (j) monitoring and surveillance. At the population level, policies to reduce harmful use of alcohol should be developed as a component of a comprehensive suicide prevention strategy, particularly within populations with high prevalence of alcohol use (WHO, 2015). In populations with lower levels of drinking, strategies such as awareness-raising can be implemented through general media campaigns, school health promotion activities or information targeted at vulnerable individuals through health professionals (Chisholm *et al*. 2004; Prince *et al*. 2007). The alcohol culture of specific regions should be considered carefully before strategies are selected in order to ensure that the strategies are effective in the context. A functioning legal system is also a prerequisite for enforcing these strategies effectively.

### Early identification and treatment

Frequently, several risk factors act cumulatively to increase a person's vulnerability to suicide. Early detection and intervention are key activities to ensuring that people receive the care they need. In this regard, all health services should incorporate suicide prevention as a core component.

The WHO mhGAP Intervention Guide in non-specialized health settings (WHO, 2010*b*) recommends comprehensively and systematically assessing everyone presenting with thoughts, plans or acts of self-harm/suicide. The guide also recommends asking any person over 10 years of age who experiences any of the other priority conditions (mental, neurological and substance use disorders), chronic pain or acute emotional distress, about his or her thoughts, plans or acts of self-harm/suicide. Asking about suicide gives the opportunity to refer to appropriate care or treatment if required (Dazzi *et al*. 2014). Protocols for clinical decision making and management are provided and tools for implementation (including a module for programme planners, situation analysis and adaptation guide, monitoring and evaluation tool, and training materials) are available for implementation in LMICs primarily. This recommended package of suicide interventions for low-resourced settings was the result of an exhaustive iterative effort by international experts, and its implementation will tell whether reduced suicides in LMICs can be demonstrated.

### Training of health workers

Education and training of health workers is needed to ensure that psychosocial support is provided to those in need and is a key way forward in suicide prevention. A large number of those who die by suicide have had contact with primary health care providers within the month prior to the suicide, and there is a growing number of LMICs where suicide awareness and skills training has been implemented in primary care services (WHO, 2014*a*). Educating health care workers to recognize depression and other mental and substance use disorders and to assess imminent risk of suicide are important for determining level of care and referral for treatment, and by that, preventing suicidal behaviour (Wasserman *et al*. 2012; Kapur *et al.* 2013). This can be implemented through the WHO mhGAP Intervention Guide in non-specialized health settings (WHO, 2010*b*). Training should take place continuously or repeatedly over years and should involve the majority of health workers in a region or country. It is important to consider local risk factors and to tailor the training programme to these in order for the programme to be successful within countries and cultures.

### Follow-up care and community support following a suicide attempt

Recently discharged patients often lack social support and can feel isolated once they leave care. Follow-up and community support have been effective in reducing suicide deaths and attempts among patients who have been recently discharged from the health care system (Luxton *et al*. 2013). Repeated follow-ups are a recommended low-cost intervention that is easy to implement; existing treatment staff, including trained non-specialized health workers, can implement the intervention and require few resources (WHO, 2010*b*). This is particularly useful in LMICs and also recognized in high-income countries. The intervention can involve the use of postcards, telephone calls or brief in-person visits (informal or formal) to make contact and encourage continued contact (Fleischmann *et al*. 2008). Involving available community support – such as family, friends, colleagues, crisis centres or local mental health centres – in aftercare is important as these can regularly monitor people and encourage treatment adherence (WHO, 2010*b*). Communities play a critical role in suicide prevention. In all countries, particularly those with limited resources, the importance of the role of communities in suicide prevention cannot be overstated, in particular in terms of support programmes for vulnerable groups. The development of integrated suicide prevention strategies, which function at the individual, family, community and societal level are the key to locally relevant and culturally appropriate suicide prevention programmes targeting the most vulnerable populations. In LMICs suicide prevention is more a social and public health objective than a traditional exercise in the mental health sector (Vijayakumar, 2004; Pearson *et al*. 2013; Malakouti *et al*. 2015). This approach harnesses community action through building community capacity, while pragmatically recognizing the finite health resources within the primary and secondary health sectors in LMICs (Vijayakumar *et al*. 2005*a*, *b*).

Having a history of previous suicide attempts is recognized as a strong predictor of subsequent death by suicide. The WHO/SUPRE-MISS (Fleischmann *et al*. 2008) – which included a number of LMICs – and other studies for high-income countries have shown that follow-up services for persons who have attempted suicide can reduce subsequent suicidal behaviour. Therefore, providing support and services for individuals who have made suicide attempts is logically a key step in preventing suicide. But in many LMICs suicide attempts seen in the emergency departments of general hospitals are treated medically and then sent home without any psychological assessment or any follow-up services. Moreover, none of the LMICs has reliable national data on the prevalence, demographic pattern, of methods of suicide attempts treated in emergency departments of general hospitals. Improving the registration of suicide attempts and developing a support network to follow-up these individuals is, perhaps, the single most practical step low-resourced LMICs can take to reduce suicides.

To conclude, the interventions discussed above are all eligible for implementation in LMICs. For universal school-based intervention programmes, there is evidence accumulating from their effectiveness in high-income countries, suggesting that they are ready to be tested in LMICs also (Aseltine *et al*. 2007; Wasserman *et al*. 2015). Other approaches, such as helplines or gatekeeper training (other than primary health care workers), are often used as best-practice approaches, but lack the conclusive evidence of effectiveness on the outcome measures of reduction in suicide or suicide attempts. Approaches like cognitive-behavioural therapy or dialectical behaviour therapy may in the present time be too costly and not feasible due to the lack of trained personnel. Community-level strategies that may have potential in preventing suicide in LMICs include utilizing the services of nongovernmental organizations, awareness raising in schools, community education around self-immolation and training of gatekeepers (Vijayakumar *et al.* 2004, 2013; Vijayakumar & Armsom, 2005; Ahmadi & Ytterstad, 2007; Wasserman *et al*. 2015; WHO, 2014*a*); however, there is need for more extensive studies to improve the evidence base around these suggestions in LMICs before they can be recommended for specific contexts.

There are indications that suicide prevention programmes that contain multiple evidence-informed interventions which are implemented simultaneously may result in reduced levels of suicide and attempted suicide (e.g. European Alliance Against Depression, Implementation of mental health service recommendations in the UK). In several culturally different countries where multi-level suicide prevention programmes had been implemented, significant reductions were observed in suicide and attempted suicide (While *et al*. 2012; Harris *et al*. 2013; Hegerl *et al*. 2013; Szekely *et al*. 2013).

## The cost and cost-effectiveness of suicide prevention efforts

In addition to evidence on the effectiveness of suicide prevention interventions, health planners and decision-makers require information on the expected costs of implementation in different settings, cultures and contexts, and also on cost-effectiveness in order to ensure that such strategies represent good value for money. Economic evaluations which take issues of context and implementation into account can help to determine whether interventions that are both effective and cost-effective in one country are feasible in others. In countries with limited resources, assessing cost-effectiveness can help determine where resources will be best allocated. For instance, an economic study of self-poisoning in Sri Lanka was able to estimate that resource needs for treatment in the country would amount to US$ 866 000 in 2004 (each treated case costing an average of US$ 32), (Wickramasinghe *et al*. 2009).

Globally, there is a lack of robust economic studies to inform planners and policy-makers of the budgetary requirements and return on investment associated with efforts to prevent suicide. A recent WHO review of suicide prevention strategies that included cost as a parameter of interest showed that two-thirds of the strategies assessed as being effective or promising were categorized as low-cost and that low cost was also closely associated with universal or selective, i.e. addressing the population as a whole or sub-populations at risk (as opposed to indicated, i.e. addressing vulnerable individuals) prevention approaches (WHO, 2010*c*). Australia's Assessing Cost-Effectiveness (ACE) in Prevention study employed a modelling approach to assess expected costs and benefits over time and assessed the comparative cost-effectiveness of a number of interventions (Vos *et al*. 2010). There are also theoretically valid upstream approaches including early childhood home visits, mentoring programmes, school-based education and community-wide prevention systems that could be preventative and cost-effective, but need further studies in both high-income countries and LMICs (WHO, 2014*a*). Such studies provide good examples of how economic analyses can be carried out and how they can inform suicide prevention strategies.

## Reaching the global target reduction of the suicide rate in countries

For national responses to be effective, a comprehensive multisectoral suicide prevention strategy, including good-quality data, is essential (Vijayakumar *et al*. 2005). A national strategy indicates a government's clear commitment to dealing with the issue of suicide. Typically, national strategies comprise a range of prevention strategies, such as means restriction, media guidelines and training for health workers. Resources should be allocated for achieving both short-to-medium and long-term objectives; there should be effective planning, and the strategy should be regularly evaluated, with evaluation findings feeding into future planning.

It is essential that governments assume their role of leadership, as they can bring together a multitude of stakeholders who may not otherwise collaborate. Governments are also in a unique position to develop and strengthen surveillance, resulting in better quality and availability of both suicide and suicide attempt data, and to provide and disseminate data that are necessary to inform action (WHO, 2014*a*).

In countries where a fully developed, comprehensive national strategy is not yet in place, this should not delay implementing targeted suicide prevention programmes since these can contribute to a national response. Regardless of the current level of implementation, all LMICs can commence on strategic actions for suicide prevention. Steps to engage stakeholders, reduce access to means, build surveillance, raise awareness, engage media, train health workers and change attitudes can be started according to each context (WHO, 2014*a*). Even if it is considered that a country is not yet ready to have a national prevention strategy, the process of consulting stakeholders about a national response often generates interest and creates an environment for change. Through the process of creating the national response, stakeholders become committed, public dialogue on stigma is encouraged, vulnerable groups are identified, research priorities are fixed, and public and media awareness are increased to ultimately reach the goal of reducing the suicide rate in countries. Particularly in countries with limited resources, the importance of communities and their support of suicide prevention programmes cannot be overstated (WHO, 2014*a*).
